# Kinetics of Chlorophyll Degradation in Japanese Maple (*Acer palmatum*) Leaves with In Situ Heating Visible and Near-Infrared Spectroscopic Monitoring

**DOI:** 10.3390/life15030335

**Published:** 2025-02-21

**Authors:** Satoru Nakashima, Hinako Yamasaki, Sumire Kanda

**Affiliations:** 1 Research Institute for Natural Environment, Science and Technology (RINEST), 3-19-20-103, Kasuga, Suita 565-0853, Osaka, Japan; 2 Department of Earth and Space Science, Osaka University, 1-1 Machikaneyama, Toyonaka 560-0043, Osaka, Japan; 3 Department of Life Science and Biotechnology, Faculty of Chemistry, Materials and Bioengineering, Kansai University, 3-3-35, Yamate-cho, Suita 564-8680, Osaka, Japan; k702074@kansai-u.ac.jp; 4 Department of Chemical, Energy and Environmental Engineering, Faculty of Environmental and Urban Engineering, Kansai University, 3-3-35, Yamate-cho, Suita 564-8680, Osaka, Japan; k580343@kansai-u.ac.jp

**Keywords:** chlorophylls, degradation, visible spectroscopy, near-infrared spectroscopy, fluorescence spectra, dehydration, color changes, first-order rates, leaf senescence, ripening, protein protection

## Abstract

Decreases in chlorophyll control the degradation of green plants during leaf senescence and fruit ripening processes. Our previous daily monitoring of the natural senescence processes of Japanese maple (*Acer palmatum*) leaves demonstrated initial slow and later fast chlorophyll (Chl) decrease rates. In this study, Chl decrease processes were monitored by in situ visible and near-infrared spectroscopy during heating of maple leaves to 30–200 °C. The initial decreases with time in the 640–720 nm band area, due mainly to chlorophyll a after the water decrease, were fitted by first-order kinetics. The obtained rate constants *k*_1_ from 200 to 60 °C showed a quasi-linear trend on an Arrhenius plot with an activation energy *E*a of 38 kJ·mol^−1^, while those from 60 to 30 °C had a different trend with an *E*a of 91 kJ·mol^−1^. Since the previous natural faster Chl decrease rates are on the extension of the higher-temperature trend, this process might occur without the protection of proteins in the photosynthetic system. On the other hand, the previous natural slower Chl decrease rates are on the extension of the lower-temperature trend, and might have protein protection.

## 1. Introduction

**Figure 1:**
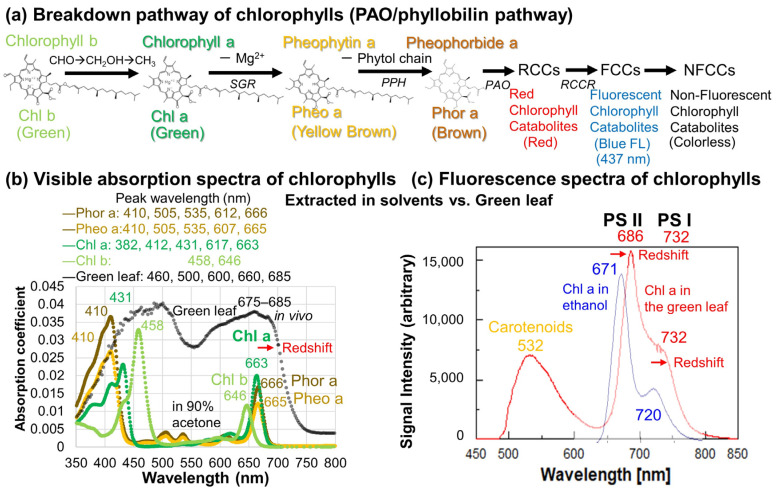
(**a**) Breakdown pathway of chlorophylls with their colors and corresponding enzymes in italics [8]; (**b**) visible absorption spectra of chlorophylls and their breakdown products (chlorophyll b, a, pheophytin a, and pheophorbide a (in 90% acetone)) from spectral data of commercially available pigment standards from [13] and that for a fresh green Japanese maple leaf (in vivo, this study) with their peak positions; (**c**) fluorescence spectra of chlorophyll a in ethanol from [14] and that measured in vivo for a fresh green Japanese maple leaf (this study) with their peak positions. Possible redshifts of absorption and fluorescence bands are indicated.

In the oxygen-producing photosynthesis of plants converting sunlight and CO_2_ to sugars, ATP, and NADP, as well as O_2_, using a combination of photosystem I (PSI) and photosystem II (PSII) in the thylakoid membranes of chloroplasts, chlorophyll a (Chl a) is the primary antenna pigment accepting sunlight around 680–700 nm, which is embedded in photosynthetic proteins [1,2]. After the photosynthetic activities of plants, generally from spring to summer, chlorophylls degrade from autumn to winter, and their breakdown pathways are considered to be from chlorophyll b (Chl b) via chlorophyll a (Chl a), pheophytin a (Pheo a), pheophorbide a (Phor a), red chlorophyll catabolites (RCCs), fluorescent chlorophyll catabolites (FCCs), non-fluorescent chlorophyll catabolites (NFCCs), and further catabolites, based on the recent literature [3,4,5,6,7,8] (Figure 1a).

The preliminary step of chlorophyll breakdown is the conversion of Chl b to Chl a by reducing aldehyde (CHO) via the alcohol (CH_2_OH) to methyl (CH_3_) branch of one of the tetrapyrrole rings assisted by Chl b reductases (NYC1/NOL) [8] (Figure 1a). This conversion of Chl b to Chl a was reported in our previous study of in situ daily monitoring of Japanese maple leaves (*Acer palmatum*) by visible spectroscopy at an early stage of their autumn senescence [9].

Chl a (green) then starts to break down, first by removal of Mg^2+^ through ion exchange with 2H^+^ from the tetrapyrrole ring, producing Pheo a (yellow brown). This process is considered to be assisted by an enzyme named stay-green (SGR), which dissociates Chl a from its binding proteins [8,10,11,12] (Figure 1a).

Pheo a (yellow brown) is converted to Phor a (brown) by removal of the phytol chain, assisted by an enzyme (PPH). Phor a is oxidatively cleaved by another enzyme (PAO) to form a type of ring-opened tetrapyrrole—red chlorophyll catabolites (RCCs). RCCs are then converted to primary fluorescent chlorophyll catabolites (FCCs) by RCC reductase (RCCR) through the reduction of a C=C double bond to a single bond. Various FCCs are transported from the chloroplast via the cytosol to the vacuole and converted to non-fluorescent chlorophyll catabolites (NFCCs) by acid-catalyzed isomerization. Most NFCCs are colorless, but some are yellow and pink [8] (Figure 1a).

Visible absorption spectra of some of these compounds [13] are shown in Figure 1b. Chl b has visible absorption bands around 458 and 646 nm in 90% acetone [13], and they have been reported to be in the range 453 nm (diethyl ether)–469 nm (methanol) and 643 nm (diethyl ether)–655 nm (pyridine) in different solvents [15]. Bands for Chl a are around 431 and 663 nm in 90% acetone [13] (Figure 1b), 429 nm (diethyl ether)–443 nm (pyridine), and 660 nm (dichloromethane)–671 nm (pyridine) [15]. For Pheo a, they are around 410 and 665 nm in 90% acetone [13], and in the range 407 nm (diethyl ether)–415 nm (benzene) and 665 nm (90% acetone)–673 nm (pyridine) [15]. The bands for Phor a are around 410 and 666 nm in 90% acetone [13], 410 nm (acetone, ethanol), and 666 nm (acetone)–668 nm (ethanol) [15]. The 663 nm bands are within the 660–673 nm range in different solvents for Chl a, Pheo a, and Phor a, and are difficult to distinguish. However, Chl b shows slightly different bands around 458 and 646 nm. On the other hand, the 433 nm band shows some differences: 410 nm (Pheo a, Phor a), 431 nm (Chl a), and 458 nm (Chl b) in 90% acetone. The absorption bands for RCCs are around 317 and 490 nm, with shoulders around 450, 520, and 580 nm [16], while those for FCCs are around 318 and 360 nm [17].

Redshifted bands of chlorophylls have been reported for in vivo spectra of photosystems, and they are considered to be bound to photosynthetic proteins [18,19]. The redshift mechanisms of the absorption bands of Chl a have recently been explained by its binding to polar charged groups or certain amino acids of proteins [20,21,22], but details remain unknown.

Fluorescence spectra of Chl a in ethanol have a peak at 671 nm and a shoulder at 720 nm [14] (Figure 1c). The fluorescence maxima have been reported at 666 nm (diethyl ether)–677 nm (pyridine) for Chl a, 646 nm (diethyl ether)–662 nm (pyridine) for Chl b, 678 nm (benzene)–686 nm (pyridine) for Pheo a, and 675 nm (ethanol) for Phor a [15]. The fluorescence bands around 670 nm are at similar positions in the 666–686 nm region for Chl a, Pheo a, and Phor a, except for Chl b, which are at slightly shorter wavelengths of 646–662 nm. RCCs have a fluorescence band around 687 nm [16], while FCCs show blue fluorescence around 437 nm [17].

Redshifted fluorescence bands of chlorophylls have been reported for in vivo spectra of photosystems, and they are considered to be bound to charged groups or certain amino acids of photosynthetic proteins [20,21,22]. It should be noted that the two fluorescence bands around 686 and 732 nm have been assigned to the photosystem II (PSII) and I (PSI), respectively [23,24] (Figure 1c).

Although these visible absorption and fluorescence spectra are similar for Chl a, Pheo a, and Phor a around 670 nm, Chl b can be recognized by the shorter-wavelength bands around 646 nm for both visible and fluorescence spectra (Figure 1b,c).

Despite the recent establishment of the breakdown pathway of chlorophylls (PAO/phyllobilin pathway) by various biochemical and biological studies [3,4,5,6,7,8] (Figure 1a), rates and time scales of chlorophyll degradation remain largely unknown.

Our previous study of in situ visible spectroscopic daily monitoring of autumn senescence processes of the Japanese maple (*Acer palmatum*) leaves during three autumn–winter seasons (2016, 2021, and 2022) [9] provided the first kinetic data for chlorophyll breakdown in nature. The leaf senescence started by initial slow decreases in Chl a (20–30 days), followed by rapid increases in anthocyanins (~20 days), and later fast decreases in Chl a (10–20 days) started after enough increases in anthocyanin. These sequential daily changes support the light-screen hypothesis of the formation of anthocyanin for the protection of leaf cells losing chlorophylls against solar radiation damage [25,26].

In this previous study, the decreases with time in the normalized 650–700 nm band areas of Chl a divided by their initial values showed two different exponential decreases corresponding to initial slow and later fast decreases in Chl a. These decreases with time in the Chl a band areas were fitted by first-order kinetics, resulting in the initial slow- and the later fast-decrease-rate constants of *k*_s_ = 2.6–3.1 × 10^−7^ s^−1^ and *k*_f_ = 1.9–5.8 × 10^−6^ s^−1^, respectively. The slower rate constants (*k*_s_) are close to that reported for Chl a decreases in the ripening of a mini tomato [27] and are also close to the Chl a decrease rate of cyanobacteria (PCC6803) with photosystems (wild type), while the faster rates (*k*_f_) are close to that of cyanobacteria without photosystems I + II (mutant) [28]. Therefore, this study proposed the following hypothesis: slow and fast chlorophyll breakdown correspond to the presence and absence of protection of chlorophylls by proteins in photosystems [9].

As mentioned above, the first step of Chl a breakdown is the removal of Mg^2+^ from the tetrapyrrole ring, and the enzyme stay-green (SGR) is considered to dissociate Chl a from bound proteins, enabling its rapid breakdown [8,10,11,12] (Figure 1a). Differences in the initial slow and later fast Chl a degradation in leaf senescence can be due to inactive and active SGR. In the natural system, this activation can be achieved by biological switching, but the details are unknown. On the other hand, enzymatic proteins can be thermally inactivated, and thermal denaturation of photosynthetic proteins has been reported to occur at over 50–60 °C [29,30,31,32]. Degradation kinetics of chlorophylls have been examined experimentally by heating green plants at temperatures from 80 to 150 °C because of needs for food processing and cooking [33,34,35].

Therefore, in this study, in order to examine Chl a decrease rates with and without thermal inactivation of proteins binding Chl a, the same Japanese maple (*Acer palmatum*) leaves before their autumn senescence were heated from 30 to 200 °C and monitored in situ by visible spectroscopy. Near-infrared spectroscopy monitoring was also conducted for evaluating water decrease processes during heating. The visible absorption band areas in the 640–720 nm range due mainly to Chl a were selected to analyze their changes with time at different temperatures. In addition, fluorescence spectrum bandshifts were measured for the heated leaves to examine Chl a binding to photosynthetic proteins, since fluorescence bands of Chl a redshifted by protein binding are expected to blueshift for Chl a dissociated from thermally denatured proteins [31,32]. The obtained Chl a degradation rates were compared with those determined in nature during the autumn senescence to examine the hypothesis that slow/fast chlorophyll breakdown corresponds to the presence/absence of protection by photosynthetic proteins [9].

## 2. Materials and Methods

### 2.1. Materials

**Figure 2:**
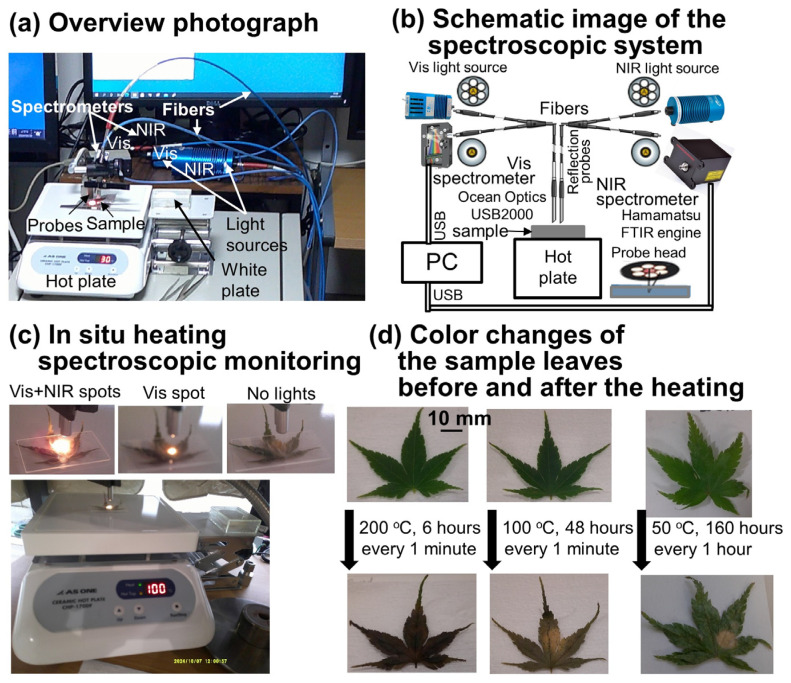
In situ heating visible (Vis) near-infrared (NIR) spectroscopic monitoring system. (**a**) Overview photograph of the system; (**b**) schematic image of the system; (**c**) close-up of a heating experiment of a Japanese maple leaf at 100 ± 1 °C on a hot plate with Vis + NIR light spots, the Vis spot only, and without lights; (**d**) color changes in the Japanese maple leaves before and after heating at 200, 100 and 50 °C. Yellow-brownish spots on leaves are photochemical damage, due mainly to the strong halogen lights on the NIR spots. The scale bar is 10 mm.

Japanese maple (*Acer palmatum*) leaves were taken from trees on the campus of Kansai University in Suita, Osaka, Japan, on the same days of the heating experiments from September to December in 2023 and 2024 before their senescence. The sizes of the leaves were 30–50 mm, with thicknesses of 0.1 to 0.3 mm (Figure 2).

### 2.2. In Situ Heating Visible Near-Infrared Spectroscopic Monitoring System

An original in situ heating visible (Vis) near-infrared (NIR) spectroscopic monitoring system was set up by the first author (Figure 2a–c).

For visible reflection spectroscopy, a light source (halogen lamp: 6.5 W) (LS-1, Ocean Optics) was connected to an optical fiber linked to a reflection probe, and applied via 6 ring fibers to the sample surface. The reflection probe top was fixed on the sample surface at a distance of about 15 to 20 mm in order not to saturate the reflected light intensities (Figure 2a,c). The visible light spot was about 5–7 mm in diameter on the sample surface. The reflected light was taken by the central optical fiber and bifurcated to a visible spectrometer (USB2000, Ocean Optics) with a 1024-channel Si detector. Reflection spectra were measured in the wavelength range of 200 to 860 nm with about 0.4 to 0.3 nm intervals. The effective wavelength range was 380–860 nm. Signals were taken for 15 milliseconds ten times in order not to saturate them (fewer than 4000 counts). First, the dark signal was taken on a white standard covered by a glass slide without the light source (dark). Second, the reference signal was taken on the white standard covered by the glass slide with the light source (reference). Third, the white signal was taken on the same white standard covered by the glass slide with the light source (sample) and the reflectance (R) was calculated by the following:

Reflectance R = (Sample − Dark)/(Reference − Dark) (1)

The reflectance spectrum of the white standard covered by the glass slide was flat, around 1 in the 380–860 nm range. Then, the reflection probe was set on the hot heating plate (ceramic hot plate CHP-170DF, AS ONE) without the glass slide and the reflectance was measured. The reflectance spectrum of the white hot plate surface was also relatively flat without significant absorption bands, but the reflectance values changed with the reflection probe distances from the surface. Finally, the sample leaf and the glass slide were placed on the center of the hot plate and heated to temperatures from 40 to 200 °C for different heating durations from 6 to 140 h. The sample reflectance spectra were measured at different intervals, (every 1, 10, 20, or 60 min) due to the spectra data size limits for data processing. These heating and measurement conditions are listed in Table 1. Since leaves are deformed during heating by losing water, the glass slide on them was used to keep them flat. Although we tried to keep the light environment of the heating experiments the same, some fluctuations due to sunlight during the day and some changes in the room lighting could not be avoided.

Near-infrared spectroscopic monitoring of the sample leaves during the heating was conducted using a miniature Fourier transform near-infrared (FT-NIR) spectrometer with MEMS technology (FTIR engine, Hamamatsu) [36] (Figure 2a,b). A high-power halogen lamp light source (HL-2000-HP, Ocean Optics: 20W) was connected to an optical fiber linked to a reflection probe (R-400-7-VIS/NIR, Ocean Optics) and applied via 6 ring fibers to the sample surface. The reflection probe top was fixed on the sample surface at a distance of about 18 to 23 mm (Figure 2a,c). The NIR light spot was about 10 mm in diameter on the sample surface. The reflected light was taken by the central optical fiber and bifurcated to the NIR spectrometer. Reflection spectra were measured in the wavelength range of 1100 to 2500 nm with about 1 to 2 nm intervals. The effective wavelength range was 1100–2300 nm. Signals were accumulated 128 times (for about 3 s). First, the reference signal was taken on the white standard covered by the glass slide with the light source (reference). Second, the white signal was taken on the same white standard covered by the glass slide with the light source (sample), and the reflectance (R) was calculated by reflectance = sample/reference. The reflectance spectrum of the white standard covered by the glass slide was flat around 1 in the 1100–2300 nm range. Then, the reflection probe was set on the hot heating plate without the glass slide and the reflectance was measured. The reflectance spectrum of the hot plate surface was also relatively flat without significant absorption bands, but the reflectance values changed with the reflection probe distances from the surface. Finally, the sample leaf and the glass slide were placed on the center of the hot plate and heated to temperatures from 40 to 200 °C for 1 to 160 h. The sample reflectance was measured every 3 s to 1 h due to the spectra data size limits for data processing. These heating and measurement conditions are listed in Table 1. Since sample leaves are deformed during heating by losing water, the glass slide on them was used to keep them flat.

### 2.3. Visible Spectroscopic Monitoring System in the Incubators

**Figure 3:**
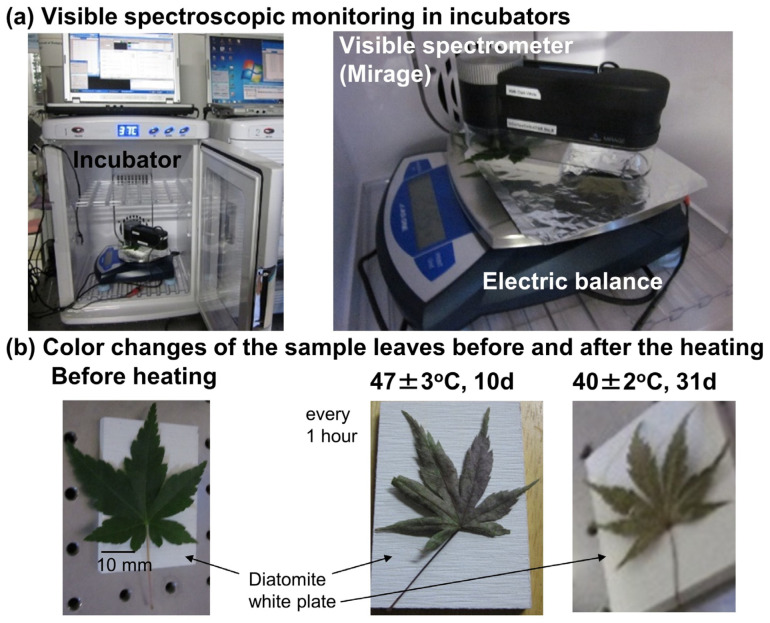
Visible (Vis) spectroscopic monitoring of leaves at low temperatures for long durations. (**a**) Photographs of Vis spectroscopic monitoring system using a spectrocolorimeter (Prismo Mirage) in an incubator at a constant temperature. (**b**) Color changes in the sample leaves before and after heating: 47 ± 3 °C for 10 days and 40 ± 2 °C for 31 days. No photochemical damage are observable, unlike Figure 2d. The scale bar is 10 mm.

For low-temperature experiments of extended duration, an original handheld visible (Vis) spectrometer (Prismo Mirage, Fuso Precision) [37] was placed in incubators with a temperature control system (Pertier electric cooling–heating system, VS-470WH, Versos) (Figure 3a). The spectrocolorimeter is composed of an LED light source, a nano-imprint grating combined with a 256-channel CMOS sensor (C10988MA-01, Hamamatsu), a wireless LAN (Wi-Fi) module, and an LCD display. A white LED light from a fluorescent plane was applied to the sample surface (about 4 mm ϕ) and collected through an optical fiber. It was dispersed by the grating and detected by the CMOS sensor. A dark spectrum (Dark) without the light source on a black surface is first measured. Then a reference spectrum (Reference) is measured on a white plate (MgO). The sample reflection spectrum was finally measured and sample reflectance values determined as a function of wavelength (nm) by Equation (1). The effective wavelength range of the sample reflectance spectra was from 400 to 760 nm.

A sample leaf was placed on a white diatomite plate on an electric balance inside the incubators (Figure 3a,b). The sample reflectance spectrum was measured every 1 h for about 10 days at 47 ± 3 °C and 40 ± 2 °C and for about 31 days at 29 ± 2 °C. These heating and measurement conditions are listed in Table 1. Temperatures, relative humidity, and weights were monitored during these periods. The temperature fluctuations were within ± 3 °C. No significant weight changes were noticed during the experimental periods. The measuring light was applied to the leaf samples only for about 2 s every 1 h, so no photochemical damage was seen (Figure 3b). The reflectance spectra of diatomite plates showed flat reflectance without noticeable absorption bands but reflectance values changed with diatomite plates.

### 2.4. Visible Near-Infrared Spectroscopic Measurements of Heated Leaves

Visible near-infrared spectra of heated leaves were measured with another original handheld Vis NIR spectrometer (Mirage Cross, Fuso Precision) with a measuring aperture of about 6 mm ϕ, as used in our previous study [27]. The effective wavelength range of the sample reflectance spectra was from 310 to 1000 nm. The two positions (Vis and NIR spots) monitored by the above in situ heating Vis NIR spectroscopic monitoring system were measured by this instrument. The color values in the CIE 1976 color space were calculated in the same way as our previous study [9]. L* is lightness, with 0 and 100 corresponding to black and white, respectively. Negative a* values correspond to green, and positive a* values correspond to red. Negative b* values correspond to blue, and positive b* values correspond to yellow. These L*a*b* values are widely used in food, agricultural, and material sciences for quantitatively describing the colors of objects and materials [9,27,37,38].

### 2.5. Microscopic Fluorescence Spectroscopy of Heated Leaves

The two positions (Vis and NIR spots) monitored by the above in situ heating Vis NIR spectroscopic monitoring system were also measured by an original fluorescence microspectrometer [38,39]. Since a fiber with a 200 μm core diameter was used for the output from the microscope (BX50, Olympus, Tokyo, Japan) to the spectrometer (SpectraPro 300i, Acton Research, Acton, MA, USA) with the CCD detector (DU420-OE, Andor, Belfast, UK), the measured spots were about 10 μm diameter with the ×20 objective lens. Fluorescence spectra were measured under the optical microscope with a ×20 objective lens by the following two methods. A 365 nm LED excitation light and a 400 nm-long path filter were used to obtain fluorescence spectra in the 400–850 nm range for 6 s 5 times. A 455 nm LED excitation light and a 500 nm-long path filter were used to obtain fluorescence spectra in the 500–850 nm range for 6 s 5 times, so 30 s in total. Since the fluorescence band around 732 nm due to PSI decays very fast, within 2 nanoseconds (ns), while the band around 686 nm due to PSII decays slowly [19], the PSII fluorescence band around 686 nm of heated leaves is expected to be mainly measured in this study (Figure 1c).

### 2.6. Determination of Absorption Spectra and Band Areas

Although diffuse reflectance values can be used as quantitative spectra for granular or powdery materials, thin leaf samples are not considered to be composed of granular structures with diffuse reflection. Moreover, negative logarithm of reflectance (absorbance) values are often taken as quantitative spectra in reflection spectroscopy of plants and food products [40,41,42,43,44]. Therefore, in this study, reflectance (R) values were converted to absorbance (Abs) values using the following Equation (2):

Absorbance (Abs) = −log Reflectance (R) = ε d *c* (2)

**Figure 4:**
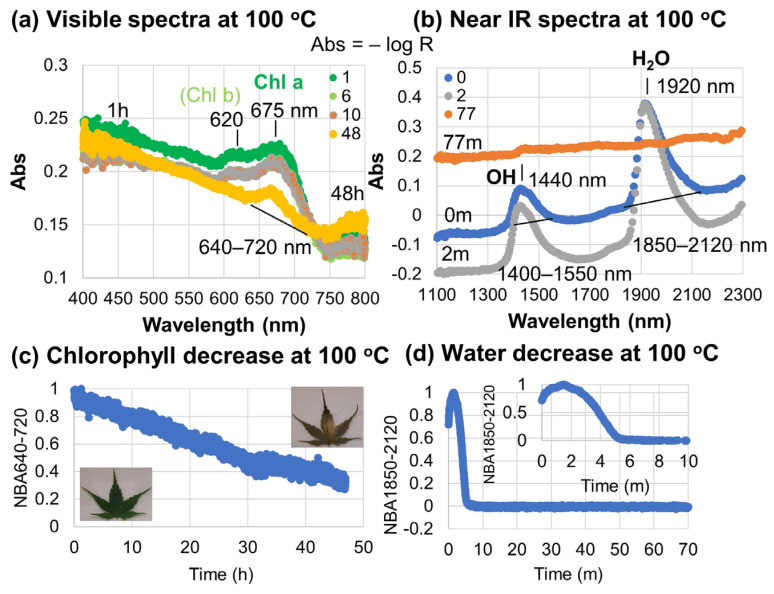
Spectral changes with time of a Japanese maple leaf heated to 100 °C for 48 h. (**a**) Representative visible absorption spectra at 1, 6, 10, and 48 h, showing bands of chlorophyll a and b; (**b**) representative near-infrared absorption spectra at 0, 2, and 77 min, showing OH and H_2_O bands; (**c**) changes with time in the normalized 640–720 nm band area, due mainly to chlorophyll a: time = 0 is shifted to the maximum value; (**d**) changes with time in the normalized 1850–2120 nm band area due to H_2_O for 70 min, with enlarged inset figure for 10 min.

This absorbance (Abs = −log R) is directly related to the concentrations (*c*) (mol·L^−1^) of species in absorption spectroscopy through Lambert–Beer’s law with the sample thickness d (cm) and the molar absorption coefficient ε (L·mol^−1^·cm^−1^) (Equation (2)). Representative absorption spectra of a sample leaf heated to 100 °C are shown in Figure 4a,b.

The visible absorption (absorbance) spectra in Figure 4a mainly show a broad band in the 590–750 nm region with the band centered around 675 nm and a shoulder around 620 nm. These bands are considered to be composed of overlapped bands of mainly chlorophyll a and b. Our previous study on the same Japanese maple leaves measured by a different handheld spectrometer used a band area of 650–700 nm for chlorophyll a [9]. Since the band shapes are slightly different from those bands due to different spectrometer responses, a band area of 640–720 nm was selected as a quantitative measure of chlorophylls in this study. The shoulder around 620 nm was not included here, because the band in the 600–640 nm region can include a major contribution from chlorophyll b (Figure 1b) and can increase at the first stage of chlorophyll breakdown due to chlorophyll b → chlorophyll a transformation, observed in our previous study [9]. Even with a lesser contribution of chlorophyll b, the band 640–720 nm includes not only Chl a (green) but also Pheo a (yellow brown) and Phor a (brown) as breakdown products (Figure 1b) during heating experiments. These points will be discussed later.

The near-infrared (NIR) spectra during heating of the Japanese maple leaf at 100 °C in Figure 4b show two absorption bands around 1440 and 1920 nm. The 1440 nm band is due to overtone of O-H stretching vibrations and the 1920 nm band is the combination of O-H stretching and H-O-H bending [37,45]. Their band areas were determined by linear baselines in the 1440–1550 nm and 1850–2120 nm regions, respectively. Changes with time in these band areas showed similar trends, and so only those for the 1850–2120 nm band area are shown in Figure 4d. They showed maxima in the first stage because of the appearance of water bubbles between the sample leaves and the glass slide in the measured spots at all the temperatures (around 1.5 min at 100 °C) by dehydration from the leaves. Therefore, the band areas were divided by their maxima to obtain normalized 1850–2120 nm band areas from 1 to 0. The water decrease process proceeded in short time periods for high-temperature experiments (about 6 min at 100 °C, Figure 4d).

In order to evaluate water content in the Japanese maple leaves, the weights of four leaves were measured before and after heating at 100 °C for 1 to 2 h (0.10 to 0.04 g, 0.08 to 0.03 g, 0.11 to 0.04 g and 0.16 to 0.06 g). Water content was 60 to 64 wt%. Therefore, this water content was considered lost during the very early stage of heating at high temperatures.

The maxima of visible absorption band area of chlorophyll a (640–720 nm) occurred around the same time as the end of dehydration. This can be explained by the relative concentration of chlorophylls by dehydration. Therefore, the normalized 640–720 nm band areas (divided by their maxima, with values from 1 to 0) were plotted against time by taking time = 0 at their maxima for all the high-temperature heating experiments, in order to analyze only the chlorophyll decrease process without the overlapping dehydration process. This normalization and time shifting enabled quantitative comparisons of chlorophyll decreases at different temperatures. An example of decreases with time in the normalized 640–720 nm band areas is shown in Figure 4c for the sample leaf heated to 100 °C for 48 h.

## 3. Results and Discussion

### 3.1. Visible Spectral Bands of the Fresh Green Japanese Maple Leaves

Visible absorption spectra converted from reflection spectra measured on a fresh green Japanese maple leaf are shown in Figure 1b (Black curve). Since five positions showed mostly identical spectra, only one representative spectrum is shown. Two broad bands around 500 and 675–685 nm were observed. The 680 nm band originates from the green color of the leaf and is considered to be mainly due to chlorophyll a (Chl a), since a 670–680 nm band is generally reported in direct spectroscopic measurements on plants including leaves [9,40,41,42,43,44]. However, the absorption maxima of Chl a in organic solvents such as 90% acetone have been reported to be around 382, 412, 431, 617, and 663 nm, with the longest band around 665 nm [13,15] (Figure 1b). The band’s maximum position (~680 nm) of the green leaf was about 10–15 nm longer than reported positions of around 665 nm for Chl a extracted in organic solvents. This band has been reported to shift to the longer wavelength (redshift) for in vivo spectra of photosystems where Chl a is bound to photosynthetic proteins [18,19]. Therefore, the redshifted band of Chl a in the present fresh green Japanese maple leaves can be understood by the binding of Chl a in the photosynthetic complexes (photosystems).

The broad 680 nm band for the green leaf had a shoulder around 620 nm (Figure 1b). This shoulder band can be due to 617 nm band of Chl a, but can include the tails of the chlorophyll b (Chl b) band around 646 nm [13,15].

Another broad band in the 350–550 nm region, centered around 500 nm with a shoulder around 430 nm, was observed for the green leaf (Figure 1b). This band can be due to carotenoids such as β-carotene with bands around 454 and 480 nm [13,43,46], but with some contributions from Chl b around 458 nm and Chl a around 431 nm [13,15,47].

The broad band around 570 nm due to anthocyanins [47] was observed for red leaves during the natural senescence of Japanese maple leaves in our previous study [9]. However, this band is not recognized in the present leaf samples, since the leaves were pre-senescent.

### 3.2. Changes in Visible Spectra and Colors of Heated Japanese Maple Leaves

**Figure 5:**
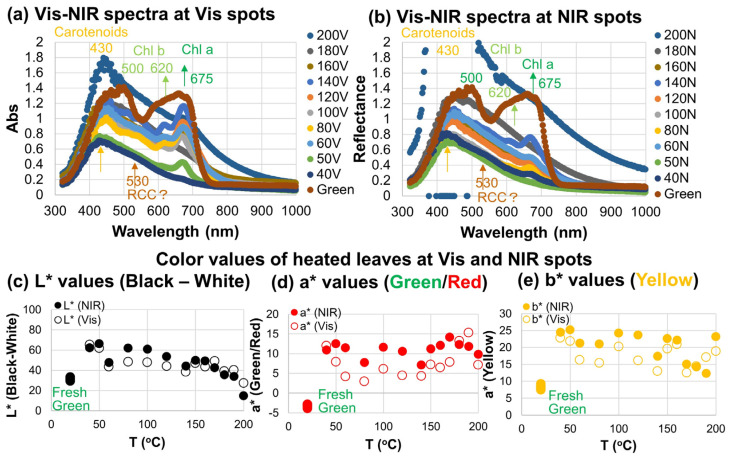
Visible near-infrared spectra of two positions (Vis and NIR spots) of Japanese maple leaves after being heated to 40–200 °C for different run durations (Table 1). (**a**) Visible absorption spectra of leaves heated to 200, 180, 160, 140, 120, 100, 80, 60, 50, and 40 °C for Vis spots and a fresh green leaf; (**b**) visible absorption spectra of leaves heated to 200, 180, 160, 140, 120, 100, 80, 60, 50, and 40 °C for NIR spots and a fresh green leaf; (**c**) L* values (black/white); (**d**) a* values (green/red) and (**e**) b* values (yellow) of leaves heated to 200, 180, 160, 140, 120, 100, 80, 60, 50, and 40 °C for Vis and NIR spots and a fresh green leaf (plotted at 20 °C). Possible band assignments are indicated with band positions (nm) in different colors.

Visible absorption spectra converted from reflection spectra measured at two positions (Vis and NIR spots) of Japanese maple leaves heated to 40–200 °C for different run durations (listed in Table 1) are shown in Figure 5a,b.

The broad 550–750 nm band centered around 675 nm and a shoulder at 620 nm observed for the fresh green leaf decreased for all the heated leaves for both the Vis and NIR spots (Figure 5a,b). Since these bands are due to Chl a and b, they are considered to have decreased by heating.

Another broad band at 350–550 nm also decreased with heating at two positions (Vis and NIR spots) of the heated leaves (Figure 5a,b). In particular, the 500 nm band decreased, while its shoulder at 430 nm remained after the heating. Therefore, the remaining 430 nm band can be attributed to carotenoids such as β-carotene and the decreasing 500 nm band to Chl b and a [13,47].

The 500 nm band and the 620 nm shoulder decreased first, and the 675 nm band due mainly to Chl a remained during the heating of leaves (Figure 5a,b). Since Chl b is converted to Chl a during the preliminary stage of chlorophyll degradation, as mentioned in the introduction (Figure 1a) [8], the components at 500 and 620 nm can be mainly assigned to Chl b. These possible assignments of different band components are indicated in Figure 5a,b.

As was discussed in our previous study of natural senescence of the same Japanese maple leaves, Chl b was first converted to Chl a, then Chl a (green) decreased, and pheophytin a (Pheo a: yellow brown) and pheophorbide a (Phor a: brown) were considered to have formed as degradation products [9] (Figure 1a). Pheo a and Phor a have been reported to have similar visible absorption bands (607 and 665 nm for Pheo a and 608 and 668 nm for Phor a) to Chl a in extracted organic solvents [13,15], and they can have contributions in the 675 band plus 620 nm shoulder (Figure 1b). Absorption coefficients of Pheo a and Phor a are small (about half of Chl a) in these bands [15]. Moreover, they can be further converted to red chlorophyll catabolites (RCCs) and fluorescent chlorophyll catabolites (FCCs) without 675 + 620 nm bands [16,17]. Since the 675 nm band and the 620 nm shoulder decreased with heating (Figure 5a,b), Chl a was considered to be converted first to Pheo a and Phor a, but further degraded to RCCs and FCCs. In fact, a weak new band around 530 nm appeared during the heating, especially for the Vis spot (Figure 5a,b), which could be due to RCCs having absorption bands in the 500–600 nm region [16].

The heated leaves changed their colors from green via yellowish brown to brown (Figure 1a, Figure 2d and Figure 3b). The color values in the CIE 1976 L*a*b* color space were determined from the reflectance spectra of the heated leaves and are shown in Figure 5c–e as a function of temperature. The five positions of the fresh green leaf are plotted at 20 °C. L* (black/white) values of heated leaves at low temperatures increased, becoming lighter, due to dehydration. With increasing temperatures, L* values decreased, becoming darker, due possibly to the formation of chlorophyll degradation products such as RCCs and FCCs with bands in the 400–600 nm region, which have large absorbance values (Figure 5a,b). For instance, the visible absorption spectrum for the NIR spot of the leaf heated to 200 °C for 6 h showed large absorbance, more than 2 (plotted as 0 absorbances), and was saturated due to dark-brown colors (Figure 2d).

Color a* (green/red) values of the five positions of the fresh green leaf were around −3, indicating green colors, while all the heated leaves showed reddish colors (Figure 5d), and a* values for the NIR spots were generally larger than those for the Vis spots, indicating more reddish colors with strong light irradiation. This reddening of heated leaves can correspond to increasing formation of red chlorophyll catabolites (RCCs) around 530 nm in the absorption spectra (Figure 5a,b).

Color b* (yellow) values of the five positions of the fresh green leaf were around 8, indicating slightly yellowish colors, while all the heated leaves showed more yellowish colors (Figure 5e), and b* values were generally larger for the NIR spots than for the Vis spots, indicating more yellowish colors with strong light irradiation. This yellowing of heated leaves can correspond to remaining carotenoids plus their degradation products around 430 nm in absorption spectra (Figure 5a,b).

### 3.3. Changes in Fluorescence Spectra of Heated Japanese Maple Leaves

A fluorescence spectrum of the fresh green Japanese maple leaf is shown in Figure 1c (red curve). The green leaf has a peak at 686 nm with a shoulder at 732 nm, and a band at 532 nm due to carotenoids. The fresh green Japanese maple leaf is expected to contain only Chl a and Chl b without degradation products (Pheo a, Phor a, RCCs, FCCs, and NFCCs). The two fluorescence bands around 686 and 732 nm have been assigned to Chl a in the photosystem II (PSII) and I (PSI), respectively [23,24]. These Chl a bands of the green leaf (686, 732 nm) are redshifted from those extracted in solvents (666–677 nm and 720 nm) [15] for about 15–12 nm (Figure 1c). Redshifted fluorescence bands of chlorophylls (681–690 nm and 732 nm) have been reported for in vivo spectra of photosystems, and they are considered to be bound to photosynthetic proteins [18,19,20,21,22,23,24]. The redshift (about 15 nm) of visible (685 nm) (Figure 1b) and fluorescence (686 nm) bands (Figure 1c) due mainly to Chl a in vivo compared to those extracted in solvents suggest a binding of Chl a to photosynthetic proteins.

**Figure 6:**
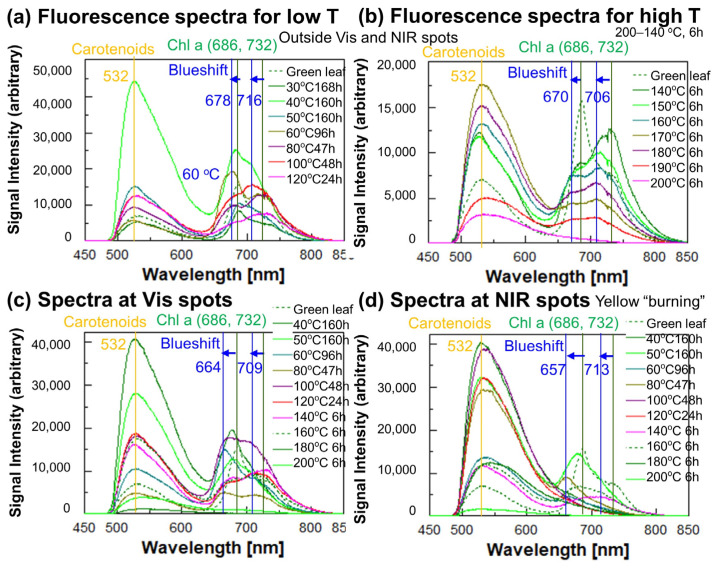
Fluorescence spectra with 455 nm excitation of Japanese maple leaves after heating to 30–200 °C for different run durations (Table 1): (**a**) at low temperatures (30–120 °C) outside the Vis and NIR spots, (**b**) at high temperatures (140–200 °C for 6 h) outside the Vis and NIR spots, (**c**) at the Vis spots, and (**d**) at the NIR spots. The spectrum for a fresh green leaf is shown as a broken green curve. Representative band maxima positions (nm) and their blueshifts from the original positions (686 and 732 nm) are indicated.

Fluorescence spectra measured at two positions (Vis and NIR spots) and outside those of Japanese maple leaves heated to 40–200 °C for different run durations (listed in Table 1) are shown in Figure 6. The spectra with 365 nm excitation show similar band shapes, but with much less signal intensity and signal-to-noise ratios, and so only the representative spectra with the 455 nm excitation are presented in Figure 6.

After heating of the green leaves, the 686 + 732 fluorescence bands showed changes in their positions and shapes (Figure 6). At low temperatures of 30, 40 and 50 °C, the 686 and 732 nm band positions did not change greatly, while at 60 °C, they blueshifted to 678 and 716 nm, respectively, outside the Vis and NIR spots (Figure 6a). At these positions, at higher temperatures of 140 to 200 °C for 6 h, they blueshifted to 670 and 706 nm, respectively, until 190 °C, while at 200 °C they become unrecognizable (Figure 6b). These blueshifts of the 686 and 732 nm bands were also observed for the Vis spots to 664 and 709 nm, respectively, and for the NIR spots to 657 and 713 nm, respectively (Figure 6c,d). These fluorescence maxima positions are summarized in Table 2.

Since the fluorescence bands for Chl b in solvents are mostly at shorter wavelengths (646–662 nm) [15] from the above bands, and Chl b is considered to already be converted to Chl a at a very early stage, the observed blueshifts might not be due to Chl b.

The fluorescence bands of pheophytin a (Pheo a) and pheophorbide a (Phor a) have been reported to be around 675–686 nm, but with much less intensity (0.06 of Chl a) [15]. The contributions of Pheo a and Phor a in the 686 nm band might be minor. They can be further converted to red chlorophyll catabolites (RCCs) and fluorescent chlorophyll catabolites (FCCs) (Figure 1a). Their fluorescence bands have been reported to be at 687 nm for RCCs [16] and at 437 nm for FCCs [17]. Although a broad fluorescence band around 520 nm in the 400–600 nm region was observed by 365 nm excitation, no clear indication of the 437 nm FCC band could be obtained. Therefore, the observed blueshifts of the 686 nm band were tentatively attributed to dissociation of Chl a from photosynthetic proteins.

The 686 nm and 732 nm fluorescence bands were assigned to photosystem II (PSII) and I (PSI), respectively [19,24], the 732 nm band due to PSI decaying rapidly, and the 686 nm band due to PSII remains [19]. In the present heating experiments of green leaves, the 686 nm band decreased first and the 732 nm band remained. Therefore, the heating and light damage to Chl a can be larger in PSII than in PSI.

Another broad fluorescence band around 532 nm was also noted for the fresh green leaf (Figure 6: broken green curves). This 532 nm band can be due to carotenoids having fluorescence bands in the 500–600 nm region [46]. The broad 532 nm band increased at low temperatures, while it decreased for high temperatures of 200 and 190 °C for 6 h (Figure 6a,b). Therefore, carotenoids might also be degraded during heating to high temperatures [48]. On the other hand, at the Vis and NIR spots, fluorescence intensities of the 532 nm band increased greatly at low temperatures for long durations (Figure 6c,d). This can be due to further photochemical degradations of carotenoids [48] with the strong halogen light irradiation at the NIR spots and the adjacent Vis spots.

**Figure 7:**
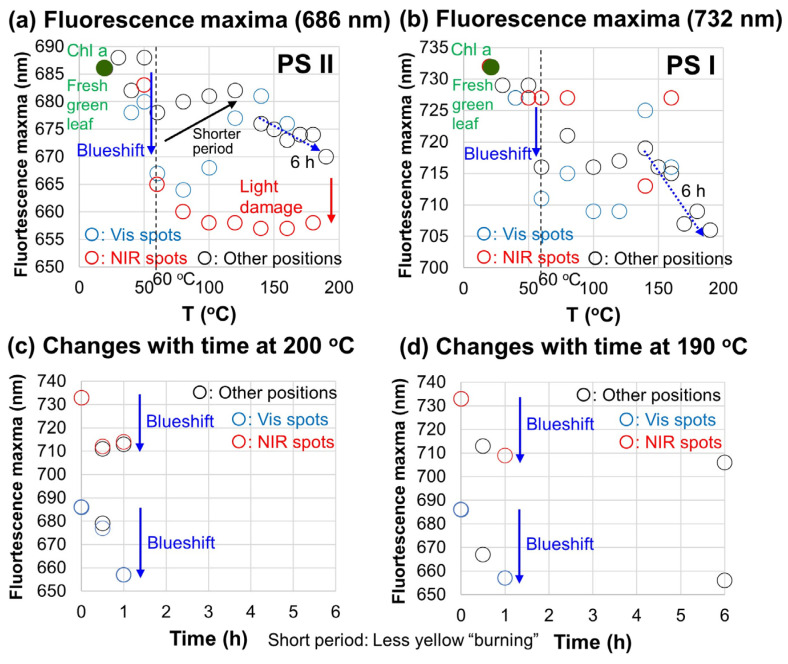
Fluorescence maxima positions with the 455 nm excitation of Japanese maple leaves after heating to 30–200 °C for different run durations (Table 2) at the Vis and NIR spots and other positions. (**a**) 686 nm band positions as a function of temperature (30–200 °C), (**b**) 728 nm band positions as a function of temperature (30–200 °C), (**c**) 686 and 728 nm band positions as a function of heating duration at 200 °C, (**d**) 686 and 728 nm band positions as a function of heating duration at 190 °C. The band positions of the fresh green leaf (686 and 728 nm) are plotted at 20 °C.

These fluorescence maxima positions are plotted for the 686 and 732 nm bands in Figure 7a,b, respectively. The 686 nm band maxima positions outside the Vis and NIR positions are around 686 nm at 30, 40 and 50 °C (Figure 7a: open black circles). Despite some data scattering due to microscopic heterogeneities of degradation, they tend to decrease at higher temperatures from 60 °C. However, from 50 to 120 °C, the band maxima tend to increase. This can be understood by shorter heating durations for higher temperatures. In fact, for the same heating duration of 6 h at 140 to 200 °C, systematic blueshifts until 670 nm for higher temperatures are recognized for the positions outside the Vis and NIR spots. (Figure 7a: open black circles). The band maxima at Vis spots show similar behavior, but with larger blueshifts until 664 nm (Figure 7a: open blue circles). At the NIR spots, the 686 nm band shows further blueshifts until 657 nm, which can be due to photochemical damage. These blueshifts of the 686 nm band can be understood by the dissociation of Chl a from protein complexes, due possibly to thermal (+photochemical) denaturation of proteins binding Chl a in PSII, which will be discussed later.

The 732 nm band positions were also around the original 732 nm at 30, 40 and 50 °C, while they generally decreased at higher temperatures from 60 °C, with some fluctuations (Figure 7b). For the same heating duration of 6 h at 140 to 200 °C, systematic blueshifts for higher temperatures were recognized for the positions outside the Vis and NIR spots (Figure 7b: open black circles). The NIR spots did not show further decreases, suggesting no significant photochemical effects on the 732 nm band due to Chl a in PSI.

In order to examine the kinetics of blueshifts of the 668 nm and 732 nm bands, two series of additional heating experiments were conducted at 200 and 190 °C for 0 to 6 h, and Vis, NIR and other positions were measured under the microscope for their fluorescence. The results are plotted in Figure 7c,d for 200 and 190 °C, respectively. The 686 nm band decreased from 686 nm to 657 nm at 200 °C for 1 h heating and to 656 nm at 190 °C for 6 h heating. The 732 nm band decreased from 733 nm to 714–711 nm at 200 °C for 0.5–1 h heating and to 706 nm at 190 °C for 1–6 h heating. These blueshifts do not show much differences for different positions (outside, Vis and NIR spots), suggesting fewer photochemical effects at short time periods. These results clearly show that the blueshift process proceeded rapidly within about 1 h at 200 and 190 °C (Figure 7c,d).

These results of blueshifts of fluorescence bands due mainly to Chl a for the 686 nm band to 670–657 nm (16–19 nm) and for the 732 nm band to 713–706 nm (18–26 nm) during the heating experiments of Japanese maple leaves at ≥60 °C can be understood primarily by the dissociation of Chl a bound to proteins in the photosystems (PSI and PSII) due to thermal denaturation of proteins [29,30,31,32], which will be discussed later. Since the strong halogen light used at the NIR spots changed the spots into yellowish colors for long heating durations, photochemical damage caused the further blueshifts for the 686 nm band for Chl a in PSII (Figure 7a) [31].

### 3.4. Chlorophyll Decreases at 200–150 °C

In order to quantify degradation of chlorophylls, as is mentioned in Section 2.6, the normalized band area 640–720 nm was selected as a quantitative measure of chlorophylls, based on our previous study on the same Japanese maple leaves [9]. The lower wavelength limit was set to 640 nm to avoid contributions from Chl b (cf. Figure 4a and Figure 5a). Although this band area can include contributions from Pheo a (665 nm) and Phor a (668 nm), which are breakdown products of Chl a, their absorption coefficients are about half that of Chl a [15], and so the normalized band area of 640–720 nm is mainly due to Chl a.

**Figure 8:**
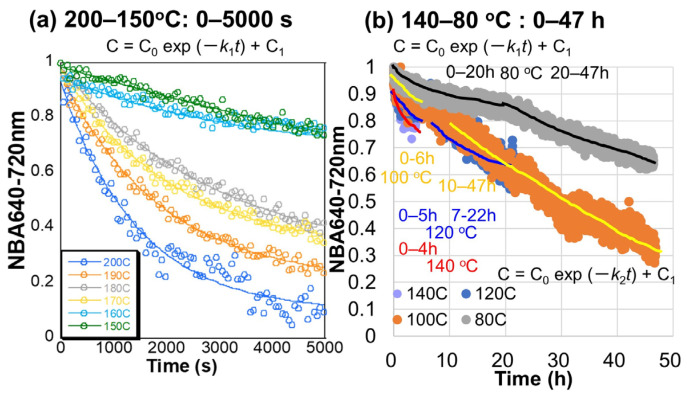
Decreases with time in the normalized 640–720 nm band areas due mainly to chlorophyll a during heating of Japanese maple leaves (Table 1): (**a**) at 200, 190, 180, 170, 160, and 150 °C for 5000 s with fitting curves; (**b**) at 140, 120, 100, and 80 °C for 50 h. Fitting curves by C = C_o_ exp (–*kt*) + C_1_ are schematically shown for the initial (rate constants *k*_1_) and the later (rate constants *k*_2_) periods.

Changes with time in the normalized 640–720 nm band areas, due mainly to Chl a, after the dehydration during heating to 200–150 °C of Japanese maple leaves every 1 min for 6 h by the in situ Vis NIR monitoring (Figure 2) are shown in Figure 8a (only for 5000 s are shown) (Table 1). The normalized 640–720 nm band areas show larger decreases at higher temperatures. Some fluctuations can be observed for several data sets. These are mainly due to fluctuations in light environments that could not be controlled during the heating experiments. The thin leaf samples might also move and deform during the dehydration process. Despite some fluctuations, decreases with time in the normalized 640–720 nm band areas appear quasi-exponential. These Chl a decreases within 2 h at 200–150 °C correspond to the initial Chl a decreases by dissociation of Chl a from photosynthetic protein complexes without much photochemical damage, based on the fluorescence results in Section 3.3.

### 3.5. Chlorophyll Decreases at 140–80 °C

Changes with time in the normalized 640–720 nm band areas after the dehydration during heating to 140–80 °C of Japanese maple leaves by the in situ Vis-NIR monitoring (Figure 2) are shown in Figure 8b. At 140, 120, 100, and 80 °C, spectral measurements were every 1 min for 6, 24, 48, and 48 h, respectively. Time intervals and durations of the heating experiments are listed in Table 1. Some fluctuations can be observed for several data sets, mainly due to fluctuations in light environments.

At 140 °C for 4 h, only one decreasing trend is observed that is similar to the higher temperatures of 200–150 °C (Figure 8a). On the other hand, two different decrease trends of the normalized 640–720 nm band areas are recognized for heating to 120, 100, and 80 °C. The first trends are for the initial periods and the second ones are for the later periods (Figure 8b). Since yellowish colors appeared for long periods at the NIR spots, the second trends can correspond to the photochemical degradations.

### 3.6. Chlorophyll Decreases at 60–40 °C

**Figure 9:**
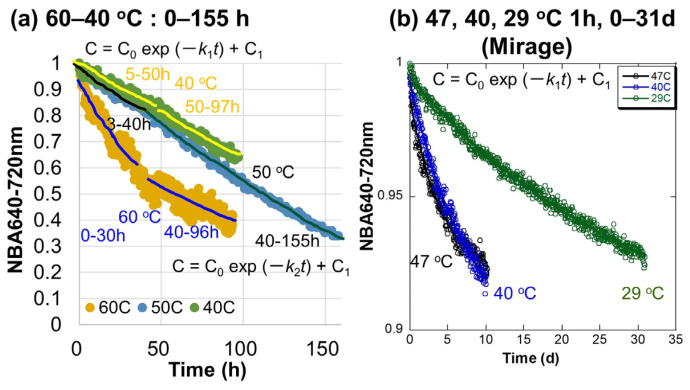
Decreases with time in the normalized 640–720 nm band areas due mainly to chlorophyll a during heating of Japanese maple leaves (Table 1): (**a**) at 60, 50, and 40 °C for 155 h; (**b**) at 47, 40, and 29 °C for 31 days. Fitting curves by C = C_o_ exp (–*kt*) + C_1_ are schematically shown for the initial (rate constants *k*_1_) and the later (rate constants *k*_2_) periods for 60, 50, and 40 °C, while for 47, 40, and 29 °C, measured by the spectrometer (Mirage) in the incubator, only one exponential decay fitting was conducted (the fitting curves are immersed in many data points).

Changes with time in the normalized 640–720 nm band areas after the dehydration during heating to 60–40 °C of Japanese maple leaves by the in situ Vis NIR monitoring (Figure 2) are shown in Figure 9a. At 60, 50, and 40 °C, spectral measurements were every 10, 60, and 20 min for 96, 160, and 160 h, respectively. Time intervals and durations of the heating experiments are listed in Table 1. Some fluctuations can be observed for several data sets, mainly due to fluctuations in light environments.

Two different decrease trends of the normalized 640–720 nm band areas are recognized similar to the heating to 120–80 °C (Figure 8b). The first trends are for the initial periods and the second ones are for the later periods. Since yellowish colors appeared for long periods at the NIR spots, the second trends can correspond to the photochemical degradations.

### 3.7. Chlorophyll Decreases at 47, 40, and 29 °C

Changes with time in the normalized 640–720 nm band areas during low temperatures (47, 40, and 29 °C) in the incubator measured by the spectrocolorimeter (Mirage) (Figure 3) of Japanese maple leaves every 1 h for long periods (10, 10, and 31 days, respectively) are shown in Figure 9b. Time intervals and durations of the heating experiments are listed in Table 1. The normalized 640–720 nm band areas show smaller decreases at lower temperatures. Fluctuations in data due to light environments are relatively small, because the sample leaves are covered by the spectrometer and placed in the incubator (Figure 3a). The decreasing trends appeared to be single and not separated into two different ones. Since only about 3 s of weak lights was applied for spectral measurements once for 1 h, the leaves were not photochemically damaged and the later photochemical chlorophyll degradation did not occur (Figure 3b).

## 4. Kinetics of Chlorophyll Degradation

### 4.1. Kinetic Analyses of Changes with Time in the Normalized 640–720 nm Band Areas

Changes with time in the normalized 640–720 nm band areas during heating of the Japanese maple leaves in Section 3.4, Section 3.5, Section 3.6 and Section 3.7 at 200–29 °C (Figure 8 and Figure 9) showed the initial and later decrease trends, which appear quasi-exponential. Therefore, these two different trends were fitted by the following equations, corresponding to first-order reaction kinetics:

C = C_0_ exp (−*kt*) + C_1_ (3)

The rate constants *k* (s^−1^) for the initial periods are named *k*_1_ and those for the later periods are *k*_2_. C_0_ and C_1_ are fitting parameters corresponding to decreased and residual values. These fitting results, together with the correlation coefficient R values, are listed in Table 1. The individual fitting curves are directly shown for the data at 200–150 °C in Figure 8a and 47, 40, and 29 °C in Figure 9b, since these data were fitted by only one Equation (3). For the data at 140–40 °C, two fitting results were obtained separately for the initial and later periods listed in Table 1 and shown schematically in Figure 8b and Figure 9a.

The fitting of the data by first-order kinetics was generally good, with correlation coefficients R larger than 0.95. However, some data fits at the later decreases were not good for the data at 120 and 60 °C, with R down to 0.83, and some data fits for the initial decreases were worse for the data at 140–100 °C, with R down to 0.53 by the fluctuating data due to changes in light environments. Despite some poor fits, most of the data could be satisfactorily represented by the first-order decrease giving the first-order rate constants *k*_1_ for the initial periods and *k*_2_ for the later periods (Table 1).

### 4.2. Changes with Temperature of the First-Order Decrease Rate Constants

**Figure 10:**
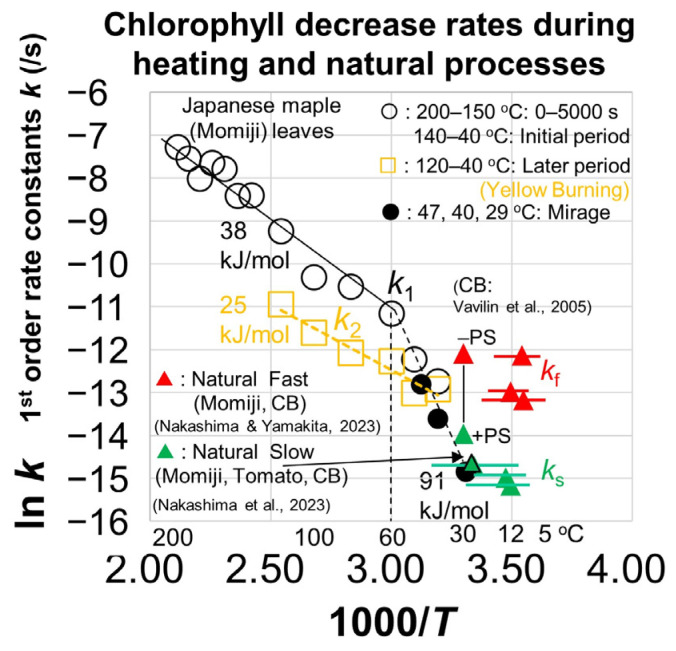
Chlorophyll decrease rate constants of Japanese maple leaves plotted in an Arrhenius diagram for initial stages (*k*_1_: open and filled black circles) and later stages (*k*_2_: open orange rectangles). The chlorophyll a decrease rate constants determined for natural senescence of Japanese maple leaves for initial slow stages (*k*_s_: filled green triangles) and for later fast stages (*k*_f_: filled red triangles) are plotted with error bars corresponding to temperature ranges [9]. The chlorophyll a decrease rate constant for the natural ripening of a mini tomato is plotted with an error bar for the temperature range (filled green triangle with a black rim) [27]. Chlorophyll a decrease rate constants for cyanobacteria with (+PS) and without (–PS) photosystems I and II at 30 °C are also shown (filled green and red triangles bound by a black line) [28].

The first-order decrease rate constants *k*_1_ for the initial and *k*_2_ for the later periods determined by the normalized 640–720 nm band areas during heating of the Japanese maple leaves are plotted in an Arrhenius diagram (Figure 10). The horizontal axis is taken as 1000/*T* for the easy conversion of gradients of the linear trends to activation energy *E*_a_ in kJ·mol^−1^. The Arrhenius equation for the temperature *T* (K) dependence of rate constants *k* is given with the Arrhenius factor *A*, *E*_a_ and gas constant R as follows:

*k* = *A* exp (−*E_a_*/R*T*) or ln *k* = *A* − *E_a_*/R*T* (4)

The first-order decrease rate constants *k*_1_ for the initial periods (open black circles with sizes representing roughly their errors) plotted in Figure 10 show a quasi-linear trend from 200 to 60 °C. The gradient of the fitted line gives *A* = 12 s^−1^ and *E*_a_ = 38 kJ·mol^−1^. On the other hand, those values at 60 to 29 °C (open and filled black circles) show a different steeper linear trend, in particular for the data at 47, 40 and 29 °C in the incubator (black filled circles). The gradient of the fitted line gives *A* = 1.7 × 10^9^ s^−1^ and *E*_a_ = 91 kJ·mol^−1^.

On the other hand, the first-order decrease rate constants *k*_2_ for the later periods (open orange rectangles) are generally smaller than *k*_1_ for the initial periods and show a different quasi-linear trend from 120 to 40 °C (Figure 10). The gradient of the fitted line gives *A* = 3.7 × 10^−2^ s^−1^ and *E*_a_ = 25 kJ·mol^−1^. Since this later chlorophyll degradation is associated with the yellowish colors at the NIR spots, this process corresponds to the photochemical degradation, which can be called “yellow burning”.

### 4.3. Different Chlorophyll Degradation Mechanisms

The above kinetic analyses of the decreases in the normalized 640–720 nm band areas during heating of the Japanese maple leaves in the Arrhenius diagram (Figure 10) suggest three different reaction mechanisms.

The later decrease rates *k*_2_ observed at 120–40 °C (open yellow rectangles) are considered to be photochemical degradation due to the strong halogen light irradiated for long hours on the NIR spots, which induced yellowing of the spots (“yellow burning”) (Figure 2d). Irradiation of lights induce the bleaching of the green color of Chl a and fluorescence quenching [49,50] and photochemical damage have been reported to occur mainly on photosystem II (PSII) (686 nm fluorescence band) [51]. In fact, our fluorescence data showed further blueshifts of the 686 nm band due to PSII, while the 732 nm band due to PSI did not show further shifts for the NIR spots with strong light illumination (Figure 7a,b). Small activation energy *E*_a_ = 25 kJ·mol^−1^ for this process suggests a mechanism with a small energy barrier. However, mechanisms for photochemical damage of photosynthetic systems are controversial and details remain unknown [51]. Therefore, we will not discuss this process further.

The absorption peak of Chl a in the fresh green Japanese maple leaves is at 675 nm (Figure 1b, Figure 4a and Figure 5a,b), which is about 15–4 nm redshifted from the reported Chl a absorption peaks in solvents from 660 (dichloromethane) to 671 nm (pyridine) [15]. The fluorescence peaks of the original green leaves are at 686 nm (Figure 6 and Figure 7, Table 2), which is about 20–9 nm redshifted from the reported Chl a fluorescence peaks in solvents from 666 (diethyl ether) to 677 nm (pyridine) [15]. In vivo absorption and fluorescence bands of Chl a in the photosystems have been reported at 675–705 nm and 680–690 nm, respectively [18,19,24,52], and redshifts have been explained by binding of Chl a to charged groups [20,53] or certain amino acids [21,22], and aggregation [52] of photosynthetic proteins. Therefore, the redshifted absorption and fluorescence peaks of the original green leaves are considered to be evidence of the binding of Chl a to photosynthetic proteins.

Thermal denaturation of proteins in photosystems has been reported to occur between 40 to 70 °C [29,30,31]. In particular, denaturation of proteins binding chlorophylls has been reported to be around 53 °C [29]. The denaturation of proteins binding Chl a results in dissociation of Chl a from protein complexes [29]. In addition, blueshift of the Chl a fluorescence band has been reported during heat-induced denaturation (680 to 670 nm at 70 °C [31]) and disassembly/degradation of Chl a-containing protein complexes (680 to 670 nm at 70 °C [32]). Therefore, the blueshift of the Chl a fluorescence bands at 686 + 732 nm during the heating experiments on the Japanese maple leaves at temperatures higher than 60 °C (Figure 6 and Figure 7) can be explained by the dissociation of Chl a from photosynthetic proteins.

The initial decrease rates *k*_1_ (open and filled black circles) showed two different linear trends, bending at 60 °C (Figure 10). The fluorescence peak at 686 nm of the heated leaves is around the same position at 30–50 °C, but blueshifted from 60 to 200 °C (Figure 6 and Figure 7). The fluorescence peaks at 140–200 °C for the same heating duration of 6 h showed increasing blueshifts for higher temperatures (Figure 7a,b). Moreover, the blueshift proceeded at 200 and 190 °C within about an hour (Figure 7c,d) in agreement with the decrease time scales of the normalized 640–720 nm absorption band areas at these high temperatures (Figure 8a). These results can be understood by the dissociation of Chl a from photosynthetic protein complexes due to thermal denaturation of Chl a-binding proteins at temperatures higher than 50 °C.

The dissociated free Chl a might be converted to degradation products such as Pheo a, Phor a, RCCs, and FCCs (Figure 1a). Although these degradation products could not be confirmed in the heated leaves, the conversion of Chl a to Pheo a by removal of Mg^2+^ from the tetrapyrrole ring is considered to be the initial step of Chl a breakdown. Since an enzyme called stay-green (SGR) is supposed to dissociate Chl a from photosynthetic proteins for accelerating Chl a breakdown in natural leaf senescence [2,10,11,12], the thermal denaturation of Chl a-binding protein is expected to induce a similar effect. This initial Chl a conversion to Pheo a by Mg^2+^ removal might be accelerated by freeing Chl a from protein binding. This Mg^2+^ removal from Chl a can be a rate-determining step of the first-order Chl a decrease reaction.

On the other hand, at low temperatures of 30–50 °C, no significant blueshifts of the fluorescence peaks at 686 and 732 nm were observed, and the initial decrease rates *k*_1_ at these temperatures (filled black circles) showed different linear trends from higher temperatures (60–200 °C) (Figure 10). This result can be explained by the stable Chl a-binding protein complexes at low temperatures, which protect Chl a from conversion to degradation products. While the Chl a decrease kinetics at 60–200 °C have an activation energy *E*_a_ of 38 kJ·mol^−1^, the *E*_a_ of 91 kJ·mol^−1^ for that at 30–50 °C is much larger, supporting slower Chl a degradation rates with a much larger energy barrier.

## 5. Comparison of Chl a Decrease Rates Between the Heating Experiments and Natural Processes

### 5.1. Chlorophyll Degradation Pathways and Kinetics: Heating Experiments and Nature

As discussed in the Introduction, the decomposition pathways of Chl a in nature have been considered to proceed from Chl a (green), via Pheo a (yellow brown), Phor a (brown), RCCs (red), FCCs (blue fluorescence), and further catabolites (Figure 1a) [8]. Although these degradation products could not be clearly confirmed only by visible or fluorescence spectroscopy, coupled with the impossibility of chemical analyses of the samples during the heating experiments, the pathways do not seem to be different from the natural ones based on their color changes, except for yellow burning by strong light irradiation (Figure 2, Figure 3 and Figure 5).

The key process of Chl a breakdown is the removal of Mg^2+^ from Chl a (green), forming Pheo a (yellow brown), which is supposed to be a rate-determining step. In nature, pheophytinase has been found to drive the removal of Mg^2+^ from Chl a, forming Pheo a [3,4,5,6,7]. Moreover, stay-green (SGR) proteins have been reported to destabilize chlorophyll–protein complexes, inducing the further degradation of chlorophylls and proteins [2,10,11,12].

Our previous study of the daily in situ monitoring of the senescence processes of Japanese maple leaves at the same positions for three periods using an original handheld visible spectrometer showed two-step exponential decreases in the normalized 650–700 nm band areas, corresponding to slow and fast first-order decrease rates, *k*_s_ = 2.6~3.1 × 10^7^ s^−1^ and *k*_f_ = 1.9~5.8 × 10^6^ s^−1^, respectively, providing some of the first kinetic data on natural chlorophyll breakdown processes [9] (Table 3). These rate constants are plotted in Figure 10, with error bars corresponding to temperature variation ranges of the monitoring periods (*k*_s_: filled red triangles; *k*_f_: filled green triangles). The slower rate constants (*k*_s_) are close to chlorophyll a decreases in the ripening of a mini tomato, monitored daily in another study by us (filled green triangle with black edge) [27] (Table 3).

Vavilin et al. (2005) [28] reported the chlorophyll decrease rates of cyanobacteria (CB: PCC6803) with (wild type: *k*_f_ +PS, green filled triangle in Figure 10) and without (mutant: *k*_s_ –PS, filled red triangle in Figure 10) photosystems I + II (Table 3). The faster *k*_f_ and slower *k*_s_ Chl a decrease rates for the Japanese maple leaves were close to CB without (–PS) and with (+PS) photosystems, respectively.

Based on these results, we hypothesized in our previous study for the Japanese maple leaves that the slower Chl a decrease rates are due to the Chl a breakdown with photosynthetic proteins protecting Chl a by its binding to proteins, while the faster decrease rates are due to the breakdown of free Chl a dissociated from proteins [9].

The present heating experiments on the same Japanese maple leaves showed two different linear trends of the initial Chl a decrease rates *k*_1_, with a bending point at 60 °C (Figure 10). Assuming that the Chl a degradation pathways are the same between the natural and heating processes, the initial decrease rates at higher temperatures with thermal denaturation can correspond to the faster Chl a decrease rates *k*_f_ in nature without the protection of Chl a-binding proteins, while those at lower temperatures with the protection of photosynthetic proteins can correspond to the slower rates *k*_s_ in nature with the protection proteins binding Chl a.

In fact, despite some data scattering with wide ranges of natural temperature variations, the natural faster rates *k*_f_ are close to the extension of the higher-temperature linear trend, while the natural slower rates are close to the extension of the lower-temperature linear trend (Figure 10). Moreover, the reported chlorophyll decrease rates of cyanobacteria (CB) [28] with photosystems I + II (*k*_f_ +PS, filled red triangle) is on the extension of the higher-temperature linear trend, while those without photosystems I + II (*k*_s_ –PS, filled red triangle) are on the extension of the lower-temperature linear trend (Figure 10). Therefore, the present hypothesis on the initial Chl a decrease rates determined for the present heating experiments of Japanese maple leaves with and without protection of Chl a-binding proteins is in agreement with natural senescence and cyanobacteria studies.

The later Chl a decrease rates *k*_2_ determined in the present heating experiments showed the third linear trend with slower rates corresponding to photochemical damage with strong lights. These can possibly explain wide ranges of faster Chl a decrease rates during natural leaf senescence, which might include effects of this photochemical damage (Figure 10).

In our heating experiments of green leaves, blueshifts of both 686 nm (PSII) and 732 nm (PSI) fluorescence bands were observed at temperatures higher than 50 °C (Figure 7). Since thermal denaturation of photosynthetic proteins associated with blueshifts (5–10 nm) of fluorescence bands around 680 nm (PSII) has been reported to occur around 60–70 °C [31,32], our fluorescence results support the above hypothesis. We conducted preliminary fluorescence measurements of various colors of Japanese maple leaves during the autumn senescence in December 2024, and found a blueshift of about 10 nm for the 686 nm band in colored leaves. Therefore, our hypothesis can also be applied to natural senescence processes.

### 5.2. Chlorophyll Degradation Mechanisms

As discussed in Section 5.1, the Chl a breakdown pathway can be the following for both natural and experimental heating processes: from chlorophyll a (Chl a), via pheophytin a (Pheo a), pheophorbide a (Phor a), red chlorophyll catabolites (RCCs), fluorescent chlorophyll catabolites (FCCs), and further catabolites (Figure 1a). The first step is the removal of Mg^2+^ from Chl a, which can be the rate-determining step of the above Chl a breakdown process. Therefore, the relatively large activation energy *E*_a_ value (91 kJ·mol^−1^) for the slower Chl a decrease at lower temperatures can correspond to the removal of Mg^2+^ from Chl a bound to photosynthetic proteins. On the other hand, the removal of Mg^2+^ from Chl a dissociated from proteins has a smaller activation energy *E*_a_ = 38 kJ·mol^−1^ with easier and faster Mg^2+^ removal.

Although there have been no detailed kinetic studies on Chl a degradation in natural plant textures, some heating experiments on processed plants have been conducted in food processing science. Canjura et al. [33] studied the degradation kinetics of chlorophylls in spinach puree at 100–145 °C and found that degradation rates were faster for Chl b than Chl a, which supports our hypothesis that the Chl a degradation is the rate-determining step. Rudra et al. [34,35] conducted heating experiments of mint and coriander puree at 80–145 °C at pH = 4.5–8.5 and determined the first-order decrease rates of Chl a with a wide range of activation energy *E*_a_ = 6.6–96 kJ·mol^−1^. The Chl a decrease rates in mint and coriander puree are faster than our data, but our *E*_a_ values (38 and 91 kJ·mol^−1^) are in the reported range.

Despite the fact that detailed mechanisms of Chl a degradation need further studies, the present results provide some of the first kinetic data on Chl a degradation rates associated with simulated thermal and photochemical damage in natural photosystems.

## 6. Conclusions

In order to examine our previous hypothesis that chlorophyll degradation rates are slow and fast with and without the protection of photosynthetic proteins [9], chlorophyll degradation processes of Japanese maple (*Acer palmatum*) leaves were monitored by in situ heating visible (Vis) and near-infrared (NIR) spectroscopy at 30–200 °C with and without the thermal denaturation of photosynthetic proteins over 50 °C. Changes with time in normalized 640–720 nm absorption band areas of chlorophyll a (Chl a) were analyzed by first-order kinetics. Fluorescence spectra by 455 nm excitation of the heated leaves were also measured.

1. The Chl a decrease rates from the normalized 640–720 nm band areas showed two different linear trends in the Arrhenius plot for 200–60 °C with an activation energy *E*_a_ = 38 kJ·mol^−1^ and for 60–30 °C with *E*_a_ = 91 kJ·mol^−1^.
2. Fluorescence maxima at 686 and 732 nm of Chl a are around the same at 30–50 °C, but blueshifted until 664 and 706 nm at higher temperatures and further to 656 nm for the NIR spots.
3. These blueshifts of fluorescence bands at temperatures higher than 60 °C can be explained by dissociation of Chl a from photosynthetic proteins due to their thermal denaturation.
4. The faster Chl a decrease rate constants *k*_f_ for the natural senescence of the Japanese maple leaves in our previous study [9] are close to the extension of the linear trend of the initial Chl a decrease rates *k*_1_ at 200–60 °C with *E*_a_ = 38 kJ·mol^−1^.
5. The slower Chl a decrease rate constants *k*_s_ for the natural senescence of the Japanese maple leaves in our previous study [9] are close to the extension of the linear trend of the initial Chl a decrease rates *k*_1_ at 60–30 °C with *E*_a_ = 91 kJ·mol^−1^.
6. Since the slower rate constant *k*_s_ is close to the Chl a decrease rate of cyanobacteria (PCC6803) with photosystems (wild type), while the faster rate *k*_f_ is close to that of cyanobacteria without photosystems (PSI + II) (mutant) [28], the former and the latter correspond to the breakdown of Chl a bound to photosynthetic proteins and dissociated from them, respectively.
7. The present in situ heating Vis and NIR spectroscopic monitoring combined with fluorescence spectroscopy can provide effective methods for simulating Chl a degradation processes in plants, especially for their kinetic behavior, but further studies are needed to examine its validity for different plant species.

## Figures and Tables

**Table 1:** Heating temperatures, durations, and spectral measurement intervals of heating experiments of Japanese maple leaves. Fitting results of the decreases in the normalized 640–720 nm band areas by first-order kinetics (C = C_0_ exp (−*kt*) + C_1_) are listed for the initial (*k*_1_) and later (*k*_2_) decreases with their data fitting periods, fitting parameters, and first-order rate constants *k*_1_ and *k*_2_.

Heating	Spectra	Data Fitting Ranges		Temperature		Fitting Parameters			Rate Constants		Fitting Parameters			Rate Constants	
Duration	Interval	Period *k*_1_	Period *k*_2_	*T* (°C)	1000/*T*	C_0_	C_1_	R	*k*_1_ (s^−1^)	ln *k*_1_	C_0_	C_1_	R	*k*_2_ (s^−1^)	ln *k*_2_
6 h	1 m	0–5000 s		200	2.113	0.835	0.096	0.980	6.89 × 10^−4^	−7.28					
6 h	1 m	0–5000 s		190	2.159	0.754	0.204	0.996	5.27 × 10^−4^	−7.55					
6 h	1 m	0–5000 s		180	2.207	0.712	0.273	0.994	3.32 × 10^−4^	−8.01					
6 h	1 m	0–5000 s		170	2.257	0.661	0.318	0.995	4.77 × 10^−4^	−7.65					
6 h	1 m	0–5000 s		160	2.309	0.227	0.734	0.970	4.15 × 10^−4^	−7.79					
6 h	1 m	0–5000 s		150	2.363	0.330	0.664	0.966	2.21 × 10^−4^	−8.42					
6 h	1 m	0–4 h		140	2.420	0.163	0.803	0.864	2.25 × 10^−4^	−8.40					
24 h	1 m	0–5 h	7–22 h	120	2.544	0.086	0.845	0.534	9.72 × 10^−5^	−9.24	0.407	0.456	0.935	1.76 × 10^−5^	−10.95
48 h	1 m	0–6 h	10–47 h	100	2.680	0.168	0.774	0.690	3.33 × 10^−5^	−10.31	0.674	0.142	0.976	9.08 × 10^−6^	−11.61
47 h	1 m	0–20 h	20–47 h	80	2.832	0.130	0.833	0.893	2.66 × 10^−5^	−10.53	0.452	0.395	0.961	5.72 × 10^−6^	−12.07
96 h	10 m	0–30 h	40–96 h	60	3.002	0.390	0.575	0.956	1.42 × 10^−5^	−11.16	0.255	0.296	0.826	4.70 × 10^−6^	−12.27
160 h	1 h	3–40 h	40–155 h	50	3.095	0.262	0.702	0.954	4.91 × 10^−6^	−12.22	0.782	0.052	0.996	2.22 × 10^−6^	−13.02
100 h	20 m	5–50 h	50–97 h	40	3.193	0.366	0.602	0.961	2.93 × 10^−6^	−12.74	0.533	0.287	0.973	2.45 × 10^−6^	−12.92
10 d	1 h	0–10 d		47	3.124	0.069	0.919	0.986	2.73 × 10^−6^	−12.81					
10 d	1 h	0–10 d		40	3.193	0.120	0.871	0.993	1.23 × 10^−6^	−13.61					
31 d	1 h	0–31 d		29	3.310	0.108	0.886	0.996	3.57 × 10^−7^	−14.85					

**Table 2:** Fluorescence band maxima (excitation by 455 nm) of heated Japanese maple leaves at Vis and NIR spots and other positions.

Maple Leaves		Fluorescence Band Maxima (nm)								
Heating		by Ex. 455 nm (6 s × 5 = 30 s)								
T (°C)	Duration	Outside Vis/NIR Spots			Vis Spots			NIR Spots		
20	Fresh	532	686	732	532	686	732	532	686	732
30	168 h	531	688	729						
40	160 h	527	682	727	528	678	727	528		
50	160 h	527	688	729	529	680	727	530	683	727
60	96 h	524	678	716	530	667	711	533	665	727
80	47 h	525	680	721	529	664	715	532	660	727
100	48 h	530	681	716	527	668	709	533	658	
120	24 h	529	682	717	529	677	709	534	658	
140	6 h	527	676	719	529	681	725	530	657	713
150	6 h	528	675	716						
160	6 h	535	673	715	530	676	716	534	657	727
170	6 h	530	674	707						
180	6 h	530	674	709	540			540	658	
190	6 h	536	670	706						
200	6 h	535			539			524		

**Table 3:** Chlorophyll decrease rates determined in the daily monitoring of autumn senescence of Japanese maple leaves [9] and ripening of a mini tomato [27]. Initial slow (*k*_s_) and later fast (*k*_f_) rate constants, data fitting period ranges, and fitting parameters are listed. Chlorophyll decrease rates for cyanobacteria (PCC6803) with and without photosystems (PS) [28] are also listed.

Sample	Spectra	Data Fitting Ranges		Temperature		Fitting Parameters			Rate Constants		Fitting Parameters			Rate Constants	
	Interval	Period *k_s_*	Period *k_f_*	*T* (°C)	1000/*T*	C_0_	C_1_	R	*k_s_* (s^−1^)	ln *k_s_*	C_0_	C_1_	R	*k_s_* (s^−1^)	ln *k_f_*
Momiji2022	1 d	24–40 d		12.9	3.496	0.408	0.783	0.589	2.60 × 10^−7^	−15.16					
	1 d		57–65 d	8.7	3.548						2.020	0.322	0.980	1.90 × 10^−6^	−13.17
Momiji2021	1 d	0–10 d		14.6	3.475	0.681	0.361	0.924	3.10 × 10^−7^	−14.99					
	1 d		30–40 d	9.2	3.542						0.803	−0.047	0.982	5.40 × 10^−6^	−12.13
Momiji2016	1 d		27–32 d	13.0	3.495						1.700	0.000	0.424	2.30 × 10^−6^	−12.98
Tomato	1 d	0–100 d		25.9	3.344	8.190	−0.590	0.988	4.07 × 10^−7^	−14.71					
Synechocystis	With PS (slow)			30.0	3.299				8.60 × 10^−7^	−13.97					
PCC6803	Without PS (fast)			30.0	3.299									5.60 × 10^−6^	−12.09

## Data Availability

All the data are presented in the present paper. Additional information will be provided upon reasonable request.
